# 

**Published:** 2010

**Authors:** Nicholas A Antao

**Affiliations:** 1001/02 Nova ‘A’ Akruti Niharika, Off N.S. Phadke Marg, Andheri East - 400 069, Mumbai, India. E-mail: narantao@gmail.com

**Figure:**
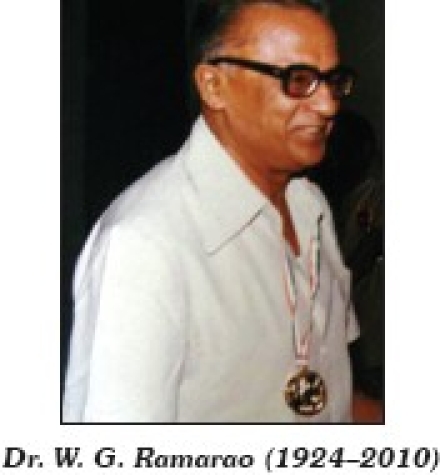
Dr. W. G. Ramarao (1924–2010)

Wunappa Gandhi Ramarao, a brilliant student from his school days, was the son of a freedom fighter. His academic excellence earned him a double FRCS from Edinburgh and London, and also the nickname Willy George or Willy Boy, thanks to an unpronounceable name to the English tongue. But, to Tara, his gynecologist wife, he was always Wunappa.

Armed with an MS (Ortho) on his return to India, he set about his goal of serving and rehabilitating the disabled. Today, the All India Institute of Physical Medicine and Rehabilitation in Mumbai stands in proud testimony of his pioneering, dedicated, selfless hard work. He worked tirelessly toward establishing the Institution and was its Director for over two decades. He worked in many academic bodies in various positions and was President of the Indian Rheumatology Association, Indian Society of Hand, Indian Association of Physical Medicine and Rehabilitation, Indian Orthopaedic Association (1985), Indian Association of Sports Medicine and Indian Foot and Ankle Society. He was one of the Founding members of the Bombay Orthopaedic Society and was also its President (1978–1979).

His work in Physical Medicine and Rehabilitation is acclaimed not only in India but also internationally. He was the national Professor of IAPMR. He was awarded the Dr. B. C. Roy National Award in 1985. He was a consultant of the World Health Organization in Cerebral Palsy for over a decade. He was also involved in setting up of the Artificial Limb Centre in Pune. He was awarded the Dr. B. N. Sinha Meritous Award in 2007 for his work in the rehabilitation of the disabled in Orthopedics. He was the proud recipient of the Vijaya Shree Award from the India International Friendship Society for enriching human life and outstanding achievement. It is no wonder then, that for all these services, he was chosen as the “Physiatrist of the Millenium.”

He was the founder fellow of the International Medical Sciences Academy and was elected Honorary fellow of the Indian Orthopaedic Association.

His special interests rested in spina bifida, cerebral palsy and adult handicap secondary to neuromuscular disorders. He had a special soft corner for the disabled and was actively involved in the Swami Vivekananda Medical Mission, surveying and tackling the problems of the adivasis and the tribals.

He was Editor of the Journal of Rehabilitation in Asia and the Indian Journal of Surgery (orthopedic section). He was twice Editor of the Indian Journal of Orthopedics. It was at the second innings that I had the priviledge to work closely with him as the Associate Editor. He was a thorough gentleman, a man of few words, who always had a message or thought to inspire others.

Dr. W. G. Ramarao served as examiner at many post graduate-level degree examinations, including MD (Physical Medicine and Rehabilitation.), MS (Ortho) and DNB (PMR). In fact, just 15 days prior to his peaceful demise (26^th^ May 2010), he had served in such capacity at one such examination.

Dr. W. G. Ramarao was always open to learning and enhancing his knowledge. He made it a point to attend the Master Series of the Bombay Orthopaedic Society to keep abreast with recent developments in orthopedics,

His passing away will be felt deeply by those who shared his passion for rehabilitation in orthopedics.

We express our deep sympathies, to his wife, Tara, daughters, Komal and Sushma, and other members of the family.

